# Supplementary figures and images for: Themis controls T cell activation, effector functions, and metabolism of peripheral CD8+ T cells
Source: Life Sci Alliance. 2023 Sep 22;6(12):e202302156. doi: 10.26508/lsa.202302156 (PMC10517225; doi:10.26508/lsa.202302156)

# Themis

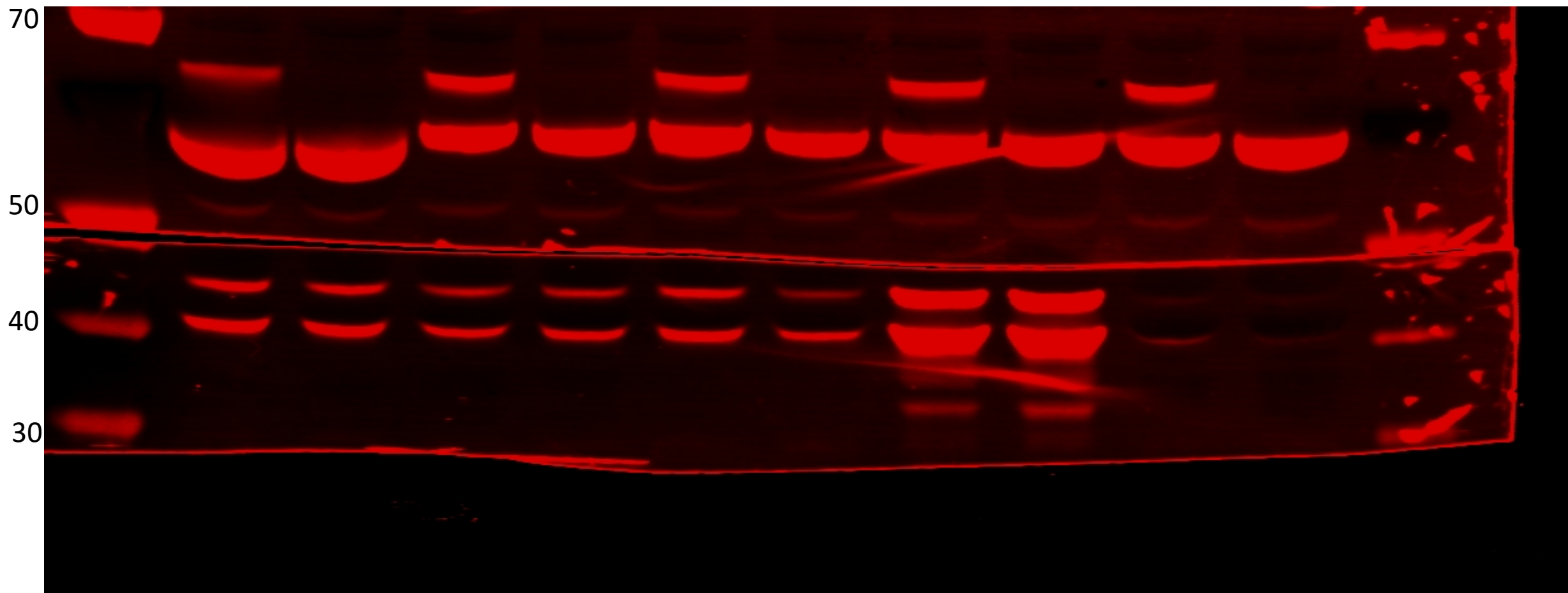

ERK1/2

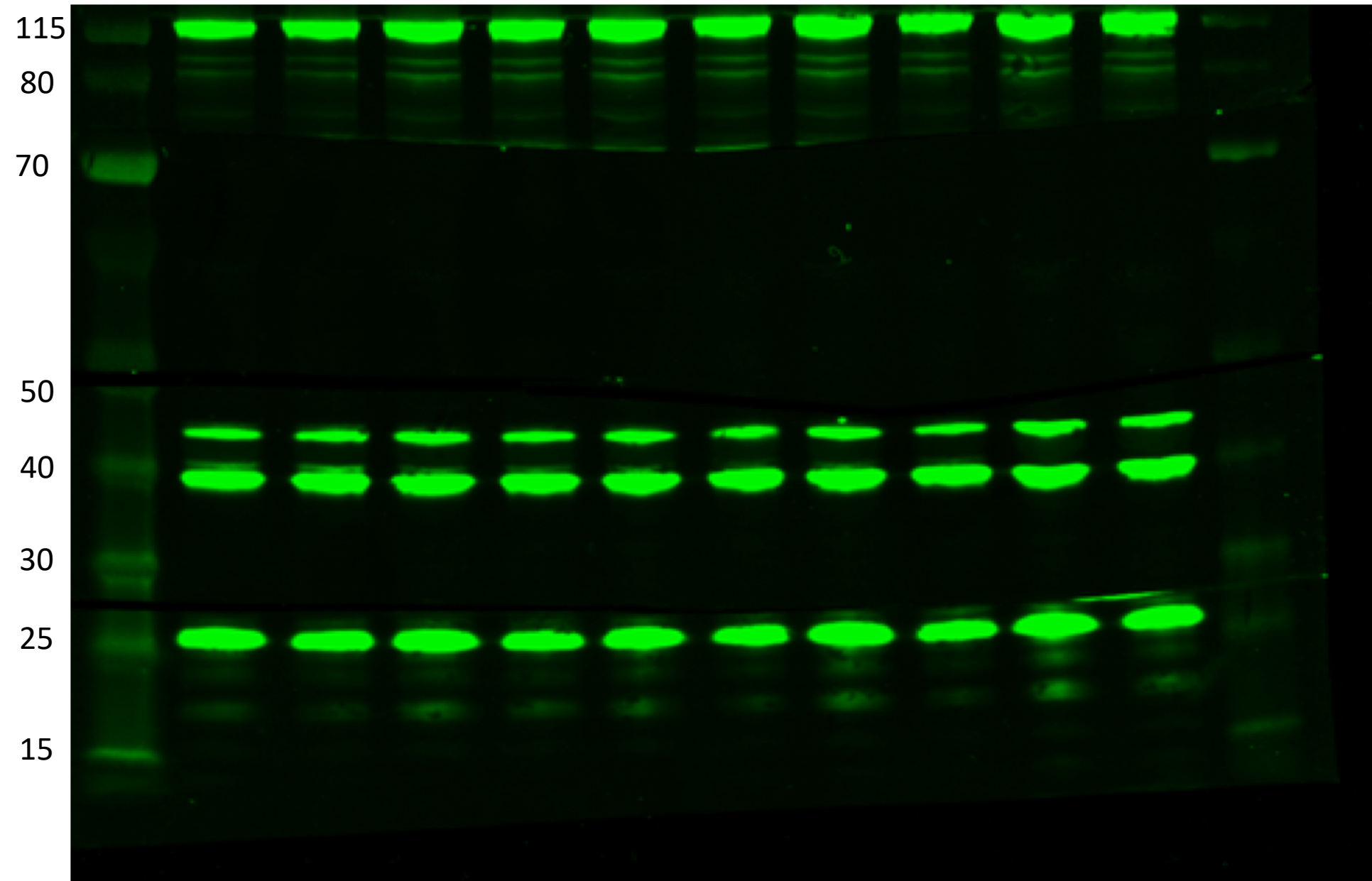

Supplement: Supplementary file 1 [file LSA-2023-02156_SdataF1.pdf]

**p-SHP1**

115

80

70

50

40

30

25

15

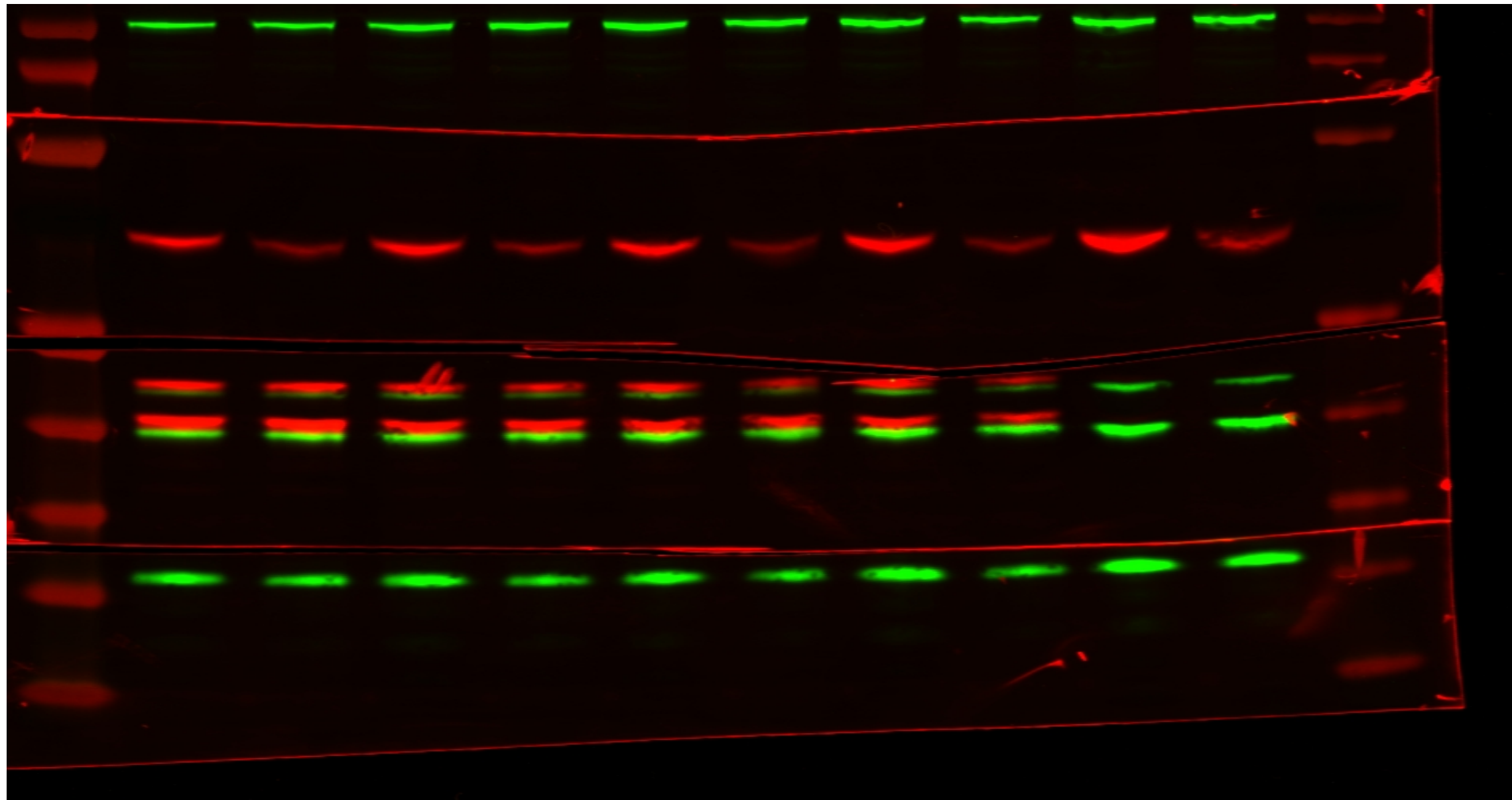

# Total SHP1

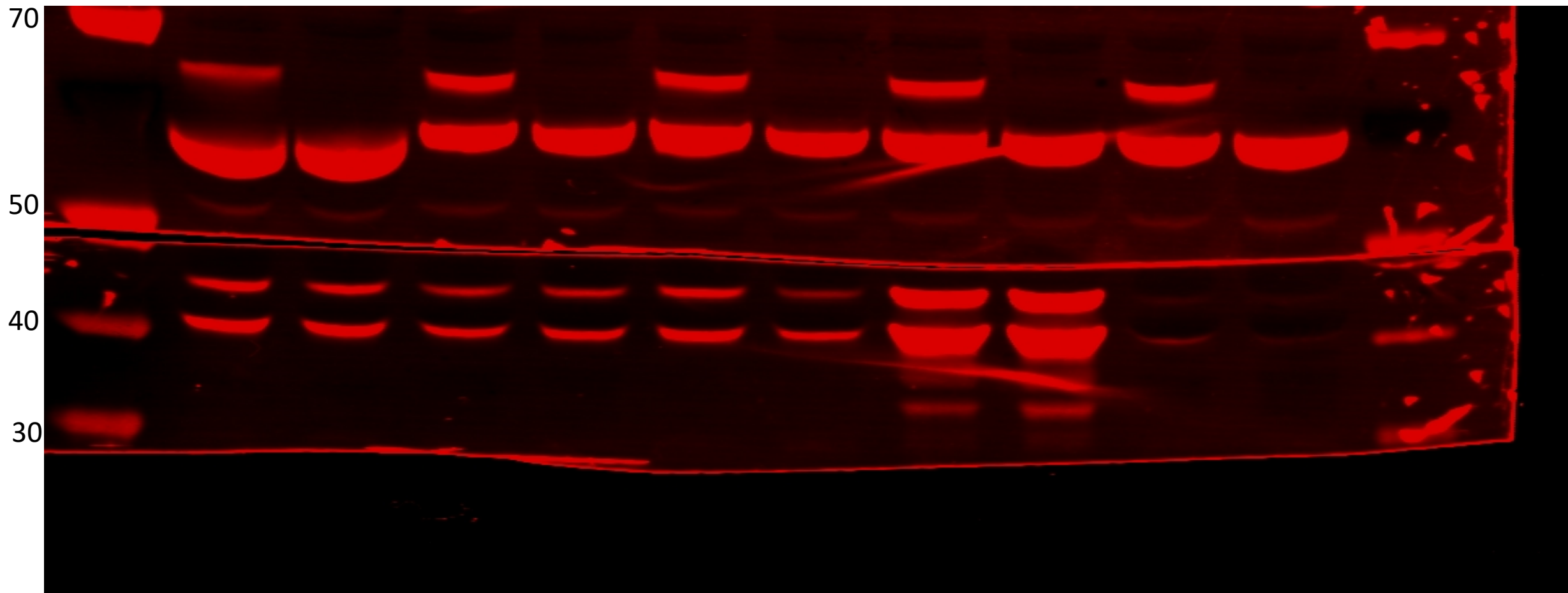

p-ERK

115

80

70

50

40

30

25

15

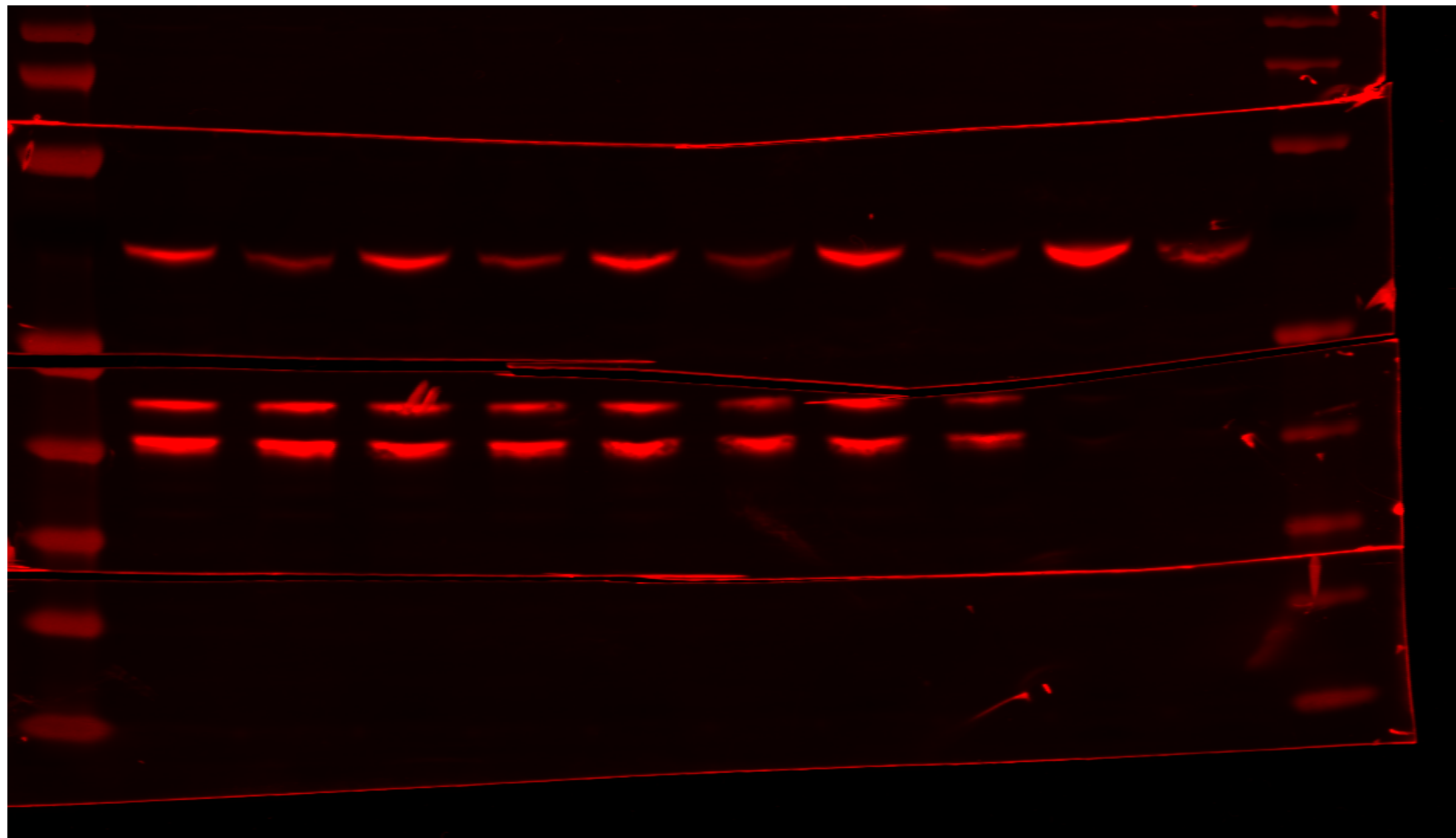

80

70

50

40

30

25

15

Supplement: Supplementary file 2 [file LSA-2023-02156_SdataF2.pdf]
