# Supplementary material for: Themis controls T cell activation, effector functions, and metabolism of peripheral CD8+ T cells
Source: Life Sci Alliance. 2023 Sep 22;6(12):e202302156. doi: 10.26508/lsa.202302156 (PMC10517225; doi:10.26508/lsa.202302156)
Supplement: Supplementary file 3 [file LSA-2023-02156_TableS1.docx]

| upregulated |  |  | downregulated |  |  |
| --- | --- | --- | --- | --- | --- |
| Hs6st1 | Pde7a | Eml4 | Ptma | Ost4 | Eif2ak2 |
| Gls | Foxo1 | Galnt1 | Rpl7a | Tmem116 | Cdc23 |
| Hspd1 | Siah2 | Fam13b | Eng | Mcm7 | Mrpl21 |
| Cul3 | Smc4 | Nrg2 | Gsn | Glcci1 | Lrp5 |
| Zc3h11a | Hdgf | Ppp2r5b | Nmi | Akr1b3 | Capn1 |
| Kdm5b | Mcl1 | Tle4 | 2700094K13Rik | Polr1a | Otub1 |
| Atp1b1 | Prss12 | Lipa | Madd | Srgap3 | Hells |
| Traf5 | Camk2d | Dntt | Cd82 | Rpl32 | Add3 |
| Pip4k2a | D4Wsu53e | Mgea5 | Nop10 | Rps9 | Was |
| Fubp3 | Clic4 | Ppp3r1 | Plcb2 | Sepw1 | Emd |
| Strbp | Nol9 | Ppp2ca | Mapkbp1 | Pglyrp1 | Pin4 |
| Myo3b | Tprgl | Hspa4 | Chchd5 | Polr2i | 9630013K17Rik |
| Pdk1 | Ski | Pdlim4 | Nop56 | Gpi1 | 2810001G20Rik |
| Trp53i11 | Gnb1 | Tagap | Vps16 | Pepd | Rap1gap2 |
| Cdan1 | Agap3 | Rftn1 | Extl2 | Nkg7 | Rpa1 |
| Gm14085 | Ccdc164 | Safb2 | H2afz | Rps11 | Nme2 |
| Eid1 | Tapt1 | Safb | 3110001D03Rik | Pgpep1l | Gm20390 |
| Trpc4ap | Ccng2 | Dirc2 | Ttc4 | Gprc5b | Cdk5rap3 |
| Srsf6 | Rnf10 | Cd47 | Ppie | 4933440M02Rik | Ndufv3 |
| Ythdf1 | Gtf2i | Ets2 | A430005L14Rik | Bola2 | Rps18 |
| Pcbp1 | Eif4h | Ptges3 | Aurkaip1 | Rasa3 | Csnk2b |
| Pvr | Sap25 | Kremen1 | Gm16008 | Ufsp2 | Tubb5 |
| Cyth2 | Arf5 | Ccdc117 | Tnfrsf18 | Gm10311 | Mrps18b |
| Cdc42se2 | Polg |  | Car12 | Nmt1 | Mrpl2 |
| Akap10 | Elavl1 |  | Ifrd2 | Gm11707 | Gm5815 |
| Vamp2 | Pkn1 |  | Rpl14 | Cd7 |  |
| Ggnbp2 | Cbfb |  | 4933406P04Rik | Acp1 |  |
| Grb2 | Rltpr |  | Themis | Hist1h1b |  |
| Mafg | Znrf1 |  | Mical1 | 2010111I01Rik |  |
| Hif1a | 1700017B05Rik |  | D630037F22Rik | Rps23 |  |
| Tubb2a | Stt3b |  | Mettl1 | Ndufs4 |  |
| Spin1 | Gtdc2 |  | Naca | Itga2 |  |
| Ctsl | Myb |  | Rps26 | Fam149b |  |
| Slc6a19 | Sec63 |  | Uqcr10 | Chchd1 |  |
| Tbc1d4 | Man1a |  | Ap1b1 | Vps28 |  |
| Fam105a | Btg1 |  | Ikzf1 | Mpst |  |
| Derl1 | Rab21 |  | Sec61g | Il2rb |  |
| Pim3 | Mdm2 |  | Npm1 | Zc3h7b |  |
| Slc38a2 | Cand1 |  | Atox1 | Tceb2 |  |

**Supplementary Table 1-** **List of up and downregulated genes overlapping between the two chronic deletion models. Please see Fig 7 legend and main text for details.**
